# Resistance to 2-Hydroxy-Flutamide in Prostate Cancer Cells Is Associated with the Downregulation of Phosphatidylcholine Biosynthesis and Epigenetic Modifications

**DOI:** 10.3390/ijms242115626

**Published:** 2023-10-26

**Authors:** José María Mora-Rodríguez, Belén G. Sánchez, Alba Sebastián-Martín, Alba Díaz-Yuste, Manuel Sánchez-Chapado, Ana María Palacín, Carlos Sánchez-Rodríguez, Alicia Bort, Inés Díaz-Laviada

**Affiliations:** 1 Biochemistry and Molecular Biology Unit, Department of Systems Biology, School of Medicine and Health Sciences, University of Alcalá, 28871 Alcalá de Henares, Madrid, Spain; josem.mora@uah.es (J.M.M.-R.); belen.sanchezg@uah.es (B.G.S.); alba.sebastian@uah.es (A.S.-M.); alba.diazy@edu.uah.es (A.D.-Y.); 2 Health Research Institute of Castilla-La Mancha (IDISCAM), 13700 Tomelloso, Ciudad Real, Spain; 3 Department of Urology, Príncipe de Asturias Hospital, 28805 Alcalá de Henares, Madrid, Spain; manuel.sanchezc@uah.es (M.S.-C.); anapalacin@hotmail.com (A.M.P.); sanchezrodriguez.car@gmail.com (C.S.-R.); 4 Department of Comparative Medicine, School of Medicine, Yale University, New Haven, CT 06519, USA

**Keywords:** LNCaP cells, flutamide, antiandrogens, dormant cells, phosphatidylcholine metabolism, resistant prostate cancer

## Abstract

In this study, we examined the metabolic adaptations of a chemoresistant prostate cancer cell line in comparison to a sensitive cell line. We utilized prostate cancer LNCaP cells and subjected them to a stepwise increase in the antiandrogen 2-hydroxy-flutamide (FLU) concentration to generate a FLU-resistant cell line (LN-FLU). These LN-FLU cells displayed characteristics of cancer stem cells, exhibited drug resistance, and showed a significantly reduced expression of Cyclin D1, along with the overexpression of p16, pointing to a proliferation arrest. In comparing the cancer stem-like LN-FLU cells to the LNCaP cells, we observed a decrease in the expression of CTP-choline cytidylyl transferase α (CCTα), as well as a decline in choline kinase, suggesting altogether a downregulation of the phosphatidylcholine biosynthetic pathway. In addition, we found decreased levels of the protein methyl transferase PRMT2 and the upregulation of the histone deacetylase Sirtuin1 (Sirt1). Analysis of the human prostate cancer samples revealed similar results in a population with high expressions of the stem cell markers Oct4 and ABCB1A1. Our findings suggest that the adaptation of prostate cancer cells to antiandrogens could induce reprogramming into stem cells that survive in a low phosphocholine metabolism and cell cycle arrest and display drug resistance.

## 1. Introduction

The concept of metabolic reprogramming was originally introduced by Hanahan and Weinberg in 2011 as a hallmark of cancer [1]. It describes the ability of tumor cells to undergo significant alterations in their metabolic pathways, enabling them to meet the heightened energy requirements necessary for rapid cell growth, invasion, and metastasis. More recently, Hanahan has proposed additional hallmarks of cancer, such as “unlocking phenotypic plasticity” and “epigenetic reprogramming” [2], that highlight the development of abnormal phenotypic capabilities during the progression of malignancy, indicating the complex and dynamic nature of cancer cells. Moreover, recent studies have emphasized the significance of metabolic reprogramming in the context of response and adaptation to therapies [3]. Understanding the metabolic response of cancer cells that have developed resistance to therapeutic drugs represents a significant ongoing challenge in cancer research [4].

In the context of prostate cancer, primary or secondary therapy resistance is a significant clinical concern, impacting approximately 30% of patients [5]. Prostate cancer typically exhibits slow growth and initially remains confined to the prostate gland. Consequently, the initial treatment approach focuses on the removal of malignant tissue. However, in cases in which this approach is ineffective, androgen withdrawal becomes a viable option. Various strategies are employed to block the androgen action on prostate growth, such as the use of androgen receptor antagonists, which have shown positive results and contributed to therapeutic advancements in the field [6]. Unfortunately, despite the success of the initial treatment, approximately one-third of prostate cancer patients experience relapse, leading to the development of a more aggressive form of cancer known as castration-resistant prostate cancer (CRPC). In recent years, there have been advancements in second-line therapies for metastatic CRPC (mCRPC), such as the use of taxanes or immune system activators [7]. However, drug resistance eventually emerges, limiting the effectiveness of these treatments. For patients who fail treatment or develop resistance, alternative therapeutic options remain unavailable at present. Therefore, understanding the underlying mechanisms driving therapy resistance in prostate cancer is crucial for the development of targeted therapies and personalized treatment approaches. 

Multiple studies have identified cancer stem cells (CSCs) as significant contributors to therapy resistance and tumor relapse [8]. These specialized subpopulations of cells express several enzymes, ATP-binding cassette (ABC) transporters, and pluripotent transcription factors that enable them to evade the effects of therapeutic interventions, leading to treatment failure and disease recurrence [9]. Emerging data have indicated that prolonged treatment with androgen deprivation therapy (ADT) can give rise to the acquisition of stemness features in prostate cancer cells [10,11], suggesting that chemotherapy could play an active role in acquired resistance [12]. This implies that chemotherapy could induce a phenotypic reprogramming in which cells develop stem cell properties and become drug resistant. Importantly, these drug-resistant cells must rewire metabolic pathways to persist under treatment [13]. However, the metabolic adaptations of the drug-resistant cells remain poorly understood. Moreover, it seems that resistant cancer cells can remain dormant for long periods of time and have unique metabolic adaptations, decreasing their energy requirements [13]. However, the roles of mitochondria, oxidative phosphorylation, and different catabolic pathways to obtain energy in the dormant refractory cells are not clearly established and may be different between different cancer types. 

Prostate cell metabolism is particularly different from that of other cell types as they inhibit the Krebs or tricarboxylic acid cycle (TCA cycle) under physiological conditions to generate substantial amounts of citrate, a component of semen [14]. Instead, prostate cells rely on lipid metabolism to sustain growth and obtain energy. It has been observed that changes in phosphatidylcholine play a role in the metabolic adaptations of prostate tumoral cells to therapy [15]. In fact, the prostate gland is one of the primary sites for the synthesis of choline and phosphocholine, which are present in high concentrations in the seminal fluid [16]. Moreover, a metabolic alteration characterized by an increased reliance on choline for fueling tumor growth and progression has been observed in advanced and untreatable castration-resistant prostate cancer [17]. Indeed, the positron emission tomography (PET) image test was developed using 18F-Choline for prostate cancer diagnosis, as this radiotracer is preferentially fixed to prostate cancer cells [18,19]. Therefore, understanding the phosphatidylcholine metabolic adaptations of drug-resistant prostate cancer cells is of critical importance in order to develop more effective treatment strategies [4].

This study aimed to investigate the adaptive phenotypic and metabolic changes that occur in prostate cells when they develop resistance to antiandrogen therapy. We focused on phosphatidylcholine metabolism as a critical adaptation for prostate cells.

## 2. Results

### 2.1. Antiandrogen-Induced Resistance in Prostate Cancer Cells: Arrest of Cell Growth and Stemness Acquirement

**Figure 1:**
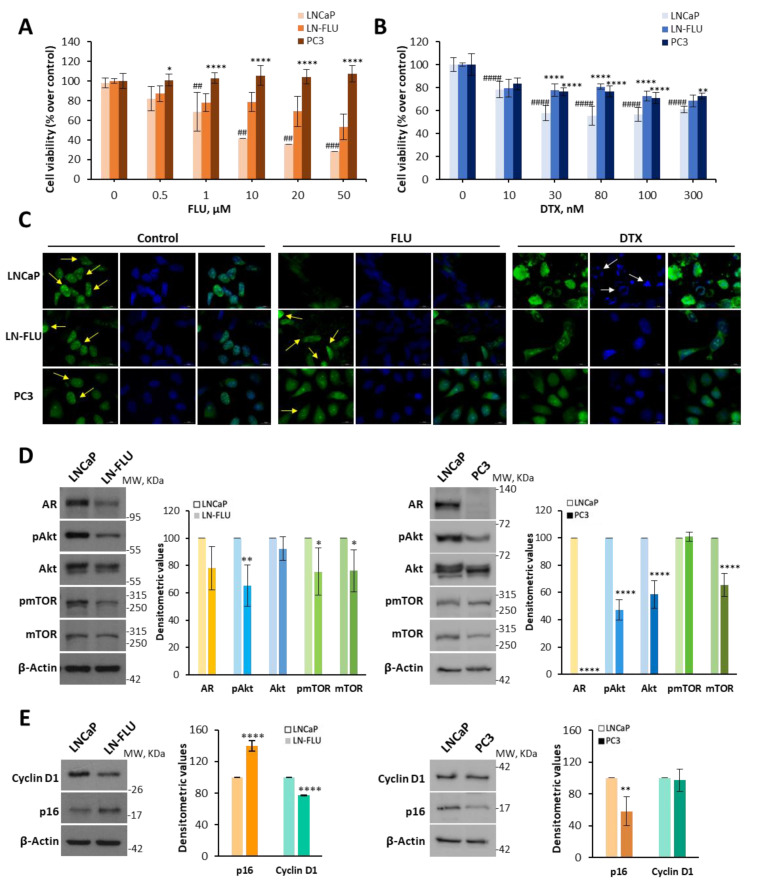
LN-FLU cells exhibit resistance to chemotherapy and cell cycle arrest. (**A**) LNCaP, LN-FLU, and PC3 cells were treated with increasing concentrations of 2-hydroxy-flutamide (FLU) or (**B**) increasing concentrations of docetaxel (DTX) for 24 h. Cell viabilities were determined via MTT assay and are expressed as percentages with respect to the control (DMSO treatment). Data are shown as the mean ± SD of at least two independent experiments. (**C**) Fluorescence images of anti-BrdU assay of cell division and proliferation. BrdU assay was carried out after cells were treated with 50 μM 2-hydroxy-flutamide (FLU) or 300 nM docetaxel (DTX) for 24 h. The incorporated BrdU was stained with anti-BrdU monoclonal antibody and then with Alexa Fluor 488-conjugated secondary antibody (green color), and the cell nuclei were stained with DAPI (blue color). Yellow arrows show proliferating cells and white arrows show apoptotic bodies. (**D**) Levels of AR, phosphorylated and total Akt, and mTOR proteins determined via Western blot. (**E**) Levels of Cyclin D1 and p16 determined via Western blot. To the right of the Western blot images are the densitometric banding analyses represented as the mean ± SD of three different experiments. ## *p* < 0.01, ### *p* < 0.001, #### *p* < 0.0001 indicates significant differences between the treated and control cells via two-way ANOVA and Tukey’s multiple comparisons test. * *p* < 0.05, ** *p* < 0.01, **** *p* < 0.0001 indicate significant differences between sensitive and resistant cells via two-way ANOVA and Sidak’s multiple comparisons test.

To investigate the adaptations of drug-resistant prostate cancer cells, we adapted the prostate tumoral cell line LNCaP, which is androgen-sensitive, to grow in the presence of the androgen receptor antagonist 2-hydroxy-flutamide (FLU) via a stepwise increase in its concentration for nine months. After this period, the cells exhibited resistance to the antiproliferative effect of FLU up to a concentration of 20 µM, with only the dose of 50 µM showing non-significant effectiveness among the tested concentrations (Figure 1A). Likewise, cells adapted to grow in the presence of FLU were less sensitive to docetaxel (DXT), compared to their parental LNCaP cells (Figure 1B). Then, we renamed the cell line LN-FLU. The resistance exhibited by the LN-FLU cells was comparable to the resistance of the prostate cancer androgen-resistant cell line PC3 (Figure 1A,B). These results indicate that after 9 months of treatment with FLU, the developed prostate cells acquired drug resistance comparable to that of classical resistant prostate cells like PC3. To further confirm the resistance of the LN-FLU cell line, we labeled proliferating cells with 5-bromo-2′-deoxyuridine (BrdU) and observed the cells via fluorescence microscopy. As observed in Figure 1C, whereas the LNCaP cells decreased proliferation and increased apoptosis when treated with FLU and DXT, in the LN-FLU and PC3 cells, still proliferating cells (yellow arrows) and less apoptosis could be observed (white arrows), corroborating the resistance of both cell lines. As expected, the DAPI (4′,6-diamidino-2-phenylindole) staining of nuclei revealed apoptotic bodies in the LNCaP cells treated with docetaxel (Figure 1C, white arrows), pointing to a docetaxel sensitivity in these cells. However, apoptotic bodies were less abundant in both the LN-FLU and PC3 cells (Figure 1C), again suggesting docetaxel resistance.

In addition to the microscopic observations, we employed a widely accepted quantitative method to evaluate the cell proliferation. We specifically utilized the CFSE (5-Carboxyfluorescein diacetate N-succinimidyl ester) probe, which diminishes as cells undergo division. FLU treatment inhibited the cell division in the LNCaP cells but not in the LN-FLU or PC3 cells (Supplementary Figure S1).

To obtain molecular evidence of the acquired resistance of the LN-FLU cells, we checked the expressions of significant proteins involved in prostate cell growth. As shown in Figure 1D, the LN-FLU cells showed less expression of the androgen receptor (AR) compared with the parental LNCaP cells, further confirming the androgen refractory state of the cells. 

**Figure 2:**
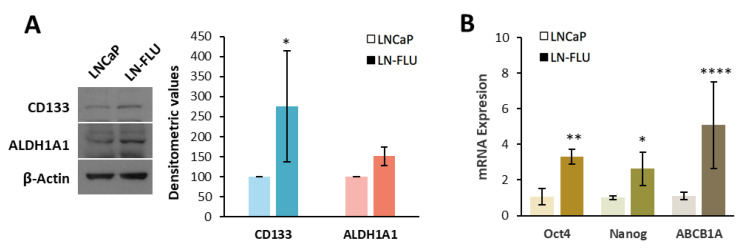
LN-FLU cells show features of cancer stem cells. (**A**) Levels of CD133 and ALDH1A1 in LNCaP and LN-FLU cells determined via Western blot. β-Actin is shown as a loading control. A representative image of three different experiments is shown. Densitometric values (mean ± SD) relative to controls are shown on the right. (**B**) Quantification of Oct4, Nanog, and ABCB1A mRNA expressions via qPCR in parental LNCaP and resistant LN-FLU cells. Actin was used as a housekeeping gene. * *p* < 0.05, ** *p* < 0.01, **** *p* < 0.0001 indicate significant differences between the LNCaP and LN-FLU cells via two-way ANOVA and Sidak’s multiple comparisons test.

The PI3K/Akt/mTOR pathway is involved in the regulation of cancer cell survival, proliferation, growth, and metabolism. In most prostate cancer cell lines, the PIP3 phosphatase PTEN, which antagonizes this pathway, is mutated and therefore the PI3K/Akt/mTOR pathway is activated. To examine the functioning of this pathway, we determined the expressions and phosphorylation levels of Akt and mTOR via Western blot. As shown in Figure 1D, in the drug-resistant LN-FLU cells, both the phosphorylation levels and overall expressions of Akt and mTOR were decreased when compared to the LNCaP cells. The results in the LN-FLU cells were similar to those observed in the androgen-resistant PC3 cells, which were used as a positive control (Figure 1D). The decrease in Akt and mTOR signaling suggests a proliferative arrest of the drug-resistant LN-FLU cells. Reduced PI3K/Akt signaling has been linked to dormancy-like phenotypes [20,21], a reversible process in which cells temporarily halt their proliferation and remain in a resting phase without undergoing division. To explore further whether the drug-resistant LN-FLU cells had undergone a dormant state, we determined the expression of Cyclin D1, which canonically drives G1/S progression by activating CDK4. Cyclin D1 was significantly decreased in the LN-FLU cells compared with the LNCaP cells (Figure 1E). Furthermore, the levels of p16, a small protein that halts the cell cycle by inhibiting CDK, exhibited an increase in the LN-FLU cells (Figure 1D). This suggests a state of slow cycling and quiescence in the LN-FLU-resistant cells, a feature not observed in PC3 (Figure 1E), as this cell line is marked by robust proliferation. Previous data have shown that dormant cells coordinate a profound growth arrest with the upregulation of self-renewal genes [22]. Then, we examined the expression levels of various stem cell markers in the quiescent cells. Our findings revealed that the LN-FLU cells exhibited an elevated expression of the stem cell-associated membrane protein CD133, which serves as a hallmark of stem cells (Figure 2A). Additionally, we observed the increased expression of the enzyme aldehyde dehydrogenase 1A1, responsible for modifying and deactivating xenobiotics within the cell (Figure 2A). Furthermore, using qPCR, we assessed the expression levels of the pluripotent transcription factors Oct4 and Nanog, along with the transporter ABCB1A, also known as glycoprotein P. Notably, ABCB1A, a well-known multidrug-resistant protein that expels drugs outside the cell, thereby promoting drug resistance, was increased in the LN-FLU cells (Figure 2B). Our results indicate that the flutamide-resistant cells entered a dormant state in which they were quiescent, while enhancing the expressions of the pluripotent transcription factors Nanog and Oct4.

### 2.2. Phosphatidylcholine Biosynthesis Is Halted in LN-FLU Drug-Resistant Cells

**Figure 3:**
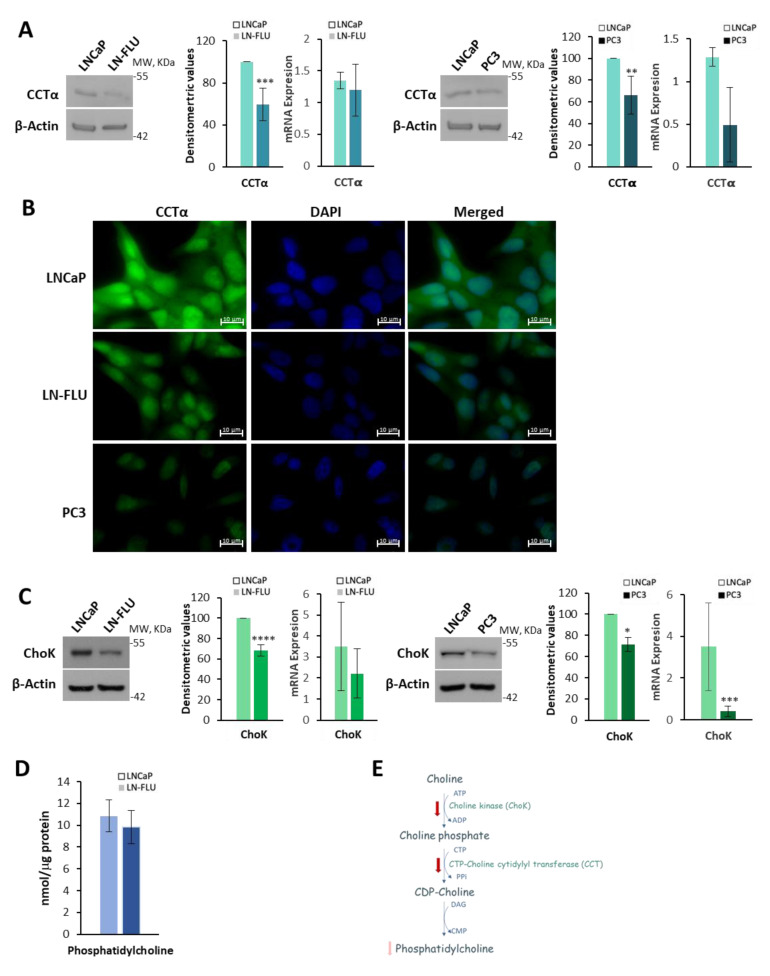
Study of the phosphatidylcholine biosynthetic pathway in LN-FLU and PC3 prostate cells. (**A**) Levels of CTP:choline cytidylyltransferase (CCTα) determined via Western blot and levels of CCTα mRNA determined via qPCR. Densitometric analysis of the Western blot bands is shown on the right of the image. Results are shown as the mean ± SD of three independent experiments. (**B**) Labeling of CCTα (green) in LNCaP, LN-FLU, and PC3 cells observed with fluorescence microscopy. Nuclei were labeled with DAPI (blue). A representative image of two different experiments is shown. (**C**) Levels of choline kinase (ChoK) determined via Western blot and levels of ChoK mRNA determined via qPCR. Densitometric analysis of the Western blot bands is shown on the right of the image. (**D**) Quantification of total phosphatidylcholine in LNCaP and LN-FLU cells using a phosphatidylcholine fluorometric assay. Phosphatidylcholine nanomoles were normalized by the amount of protein. (**E**) Diagram of phosphatidylcholine biosynthesis showing the metabolites and enzymes that we found down (red arrows) in LN-FLU cells compared to LNCaP cells. * *p* < 0.05, ** *p* < 0.01, *** *p* < 0.001 and **** *p* < 0.0001 indicate significant differences between LNCaP and LN-FLU cells via two-way ANOVA and Sidak’s multiple comparisons test.

It has been described that changes in phosphatidylcholine (PC) metabolism are part of an adaptive program that is triggered in response to stressful conditions [23]. Choline and phosphorylcholine are highly concentrated in the seminal fluid, making the prostate one of the primary tissues involved in their synthesis. Moreover, increased PC has been observed in prostate cancer, and it has been suggested that it could be a marker of prostate cancer aggression [24,25]. Nevertheless, decreased PC levels have been shown in prostate samples of patients who underwent PSA recurrence, and reduced PC levels could potentially serve as a predictive marker for biochemical recurrence following radical prostatectomy [26]. We previously employed a metabolomic approach to investigate the adaptive response associated with chemotherapy resistance [27]. In addition to observing alterations in fatty acid oxidation and ADP-ribosylation [27], we also observed a decrease in the phosphocholine levels, although this finding was not validated. Thus, here we decided to go deeper into PC synthesis, and we determined the levels of the enzyme CTP:phosphocholine cytidylyltransferase alpha (CCTα), which catalyzes the rate-limiting step in the biosynthesis of PC via the Kennedy pathway. As shown in Figure 3A, the levels of the CCTα enzyme determined via Western blot, and the levels of CCTα mRNA determined via qPCR, were decreased in the LN-FLU cells compared to the LNCaP cells. Likewise, both CCTα protein and mRNA were reduced in the androgen-refractory PC3 cells, suggesting that the decrease in phosphatidylcholine (PC) synthesis might be a common mechanism in prostate-resistant cells.

To further corroborate this finding, we performed the immunofluorescence of the LNCaP, LN-FLU, and PC3 cells using anti-CCTα as a primary antibody. The results show a reduction in fluorescence labeling in the LN-FLU cells and PC3 cells compared to the LNCaP cells (Figure 3B). However, changes in the subcellular localization of CCTα could not be observed. Then, we determined the expression of choline kinase (ChoK), the enzyme that catalyzes the phosphorylation of choline to form phosphorylcholine (PCho) in the presence of ATP and magnesium [28], which is the first step in the biosynthesis of phosphatidylcholine from choline. As shown in Figure 3C, the levels of ChoK were lower in the resistant LN-FLU cells and PC3 cells than in the androgen-sensitive LNCaP cells. Then, by using a coupled enzyme reaction, we measured the cell phosphatidylcholine content in the LNCaP cells as well as in the LN-FLU cells. The results in Figure 3D show a decrease in PC in the LN-FLU cells, although it was not statistically significant. These results indicate a drop in phosphatidylcholine biosynthesis (summarized in Figure 3E) when cells are adapted to grow in the presence of antiandrogens.

### 2.3. Protein Methylation and Deacetylation 

**Figure 4:**
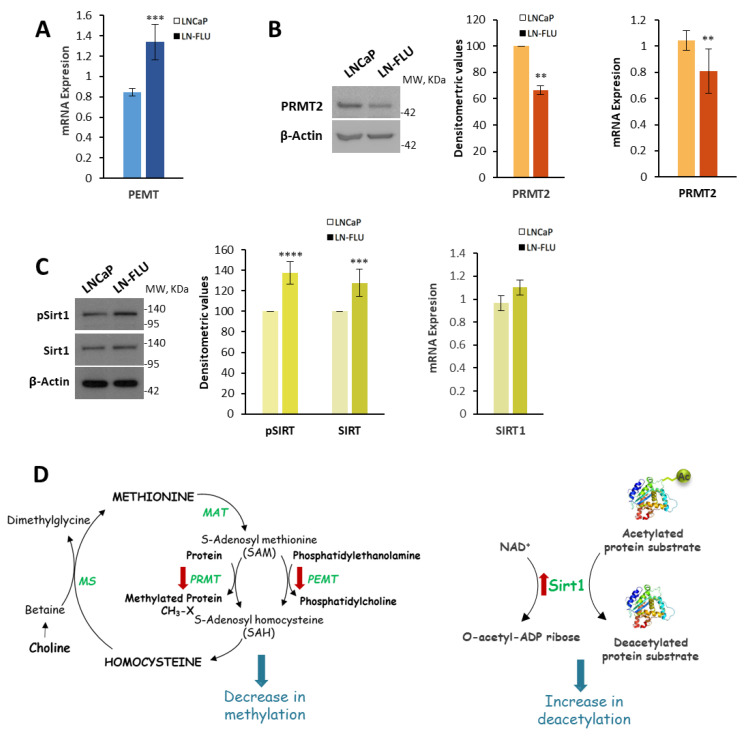
Enzymes involved in protein methylation and deacetylation in prostate LN-FLU cells. (**A**) Levels of phosphatidylethanolamine methyl transferase (PEMT) mRNA expression were determined via qPCR using Actin as a housekeeping gene. (**B**) Levels of protein arginine N-methyl transferase 2 (PRMT2) expression determined via Western blot (left) and qPCR (right). (**C**) Levels of phosphorylated and total forms of Sirtuin1 (Sirt1) determined via Western blot and densitometric analysis of the Western blot bands from three independent experiments. On the right, levels of Sirt1 mRNA were determined via qPCR using Actin as a housekeeping gene. Data show the mean ± SD of at least three different experiments. ** *p* < 0.01, *** *p* < 0.001 and **** *p* < 0.0001 indicate significant differences between LNCaP and LN-FLU cells via two-way ANOVA and Sidak’s multiple comparisons test. (**D**) Diagram of the reactions catalyzed by the enzymes analyzed showing the metabolites and enzymes that we found different (red arrows) between LN-FLU cells and LNCaP cells.

In addition to the CDP-choline (Kennedy) pathway for PC synthesis, certain tissues employ an alternative pathway for PC production. This pathway involves the sequential methylation of phosphatidylethanolamine (PE) to generate PC through three consecutive methylation reactions. In these reactions, S-adenosylmethionine (SAM) acts as the methyl group donor, and the enzyme that catalyzes the three steps is phosphatidylethanolamine methyl transferase (PEMT). As a result, PC metabolism is intricately connected to the methylation pathways, as SAM serves as the primary donor of the methyl groups in various methylation reactions throughout the cell. To evaluate whether this pathway was modified, we checked the expression of PEMT and found that it was elevated in the resistant LN-FLU cells (Figure 4A). This finding suggests a higher consumption of SAM that could impact other methylation reactions.

We then determined the expression of the protein arginine N-methyltransferase 2 (PRMT2) that catalyzes arginine methylation in proteins, including those involved in epigenetic regulation [29]. The protein as well as mRNA levels of PRMT2 were decreased in the LN-FLU cells compared to the LNCaP cells (Figure 4B), suggesting a reduction in protein methylation. These results are in line with previous data showing a reduction in PRMT2 in prostate cancer [30] and suggest that the change in the methylation reactions in the resistant cells could impact gene expression via epigenetic regulation. 

Another fundamental pattern of epigenetic modification is histone acetylation, which plays a pivotal role in controlling gene expression. Sirtuin1 (Sirt1) is a NAD+-dependent histone deacetylase that has been strongly connected with the stress response and an extended life span. We then analyzed the levels and phosphorylation of Sirt1, as well as the mRNA levels, and we found that they were increased in the LN-FLU drug-resistant cells compared to the LNCaP cells (Figure 4C), further supporting the epigenetic change in the prostate LN-FLU cells. Figure 4D shows the main metabolic pathways in which PRMT, PEMT and Sirt1 are involved.

Thus, the tumor cell microenvironment, including the presence of flutamide in the medium, could induce an epigenetic regulation that triggers a phenotypic transition towards metabolically dormant cells with stem cell-like properties, characterized by low metabolic activity and drug resistance.

### 2.4. Patients’ Baseline Characteristics

To assess the clinical significance of our findings, we conducted analyses on human prostate samples. The prostates of 15 patients scheduled for radical prostatectomy were used for this study. The baseline demographic and clinical characteristics of these patients are summarized in Table 1. Patients had an average age of 66.8 years ranging from 55 to 75 years and an average weight between 64 and 112 Kg. The Body Mass Index (BMI) ranged from 22.9 to 37.9 kg/m^2^. The Gleason’s grade was determined by a pathologist from the overall prostate piece and varied from 3 + 3 to 4 + 5. The patient’s PSA values fluctuated from 1.23 to 43 ng/mL with a median of 7.2 ng/mL.

### 2.5. CCTα, PRMT2, and Sirt1 in Human Prostate Cancer Biopsies

**Figure 5:**
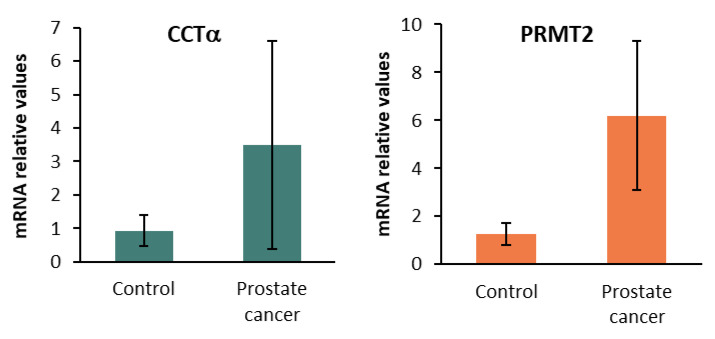
Levels of CTP:choline cytidyltransferase α (CCTα) and protein arginine N-methyl transferase 2 (PRMT2) in human prostate cancer simples. Expressions of CCTα and PRMT2 mRNA were determined via qPCR. The data show the relative mRNA expression to Actin, which was used as a housekeeping gene. Data represent the mean ± SD of control human samples (*n* = 5) and prostate cancer samples (*n* = 15).

After the RNA isolation of the samples, we determined the CCTα, PRMT2, and Sirt1 mRNA in the human prostate lysates via qPCR. The results indicated that CCTα and the methyltransferase PRMT2 were increased in the prostate cancer samples compared to the adjacent normal prostate tissue considered as the control (Figure 5). However, there was considerable variability in the expression levels among the different samples.

**Figure 6:**
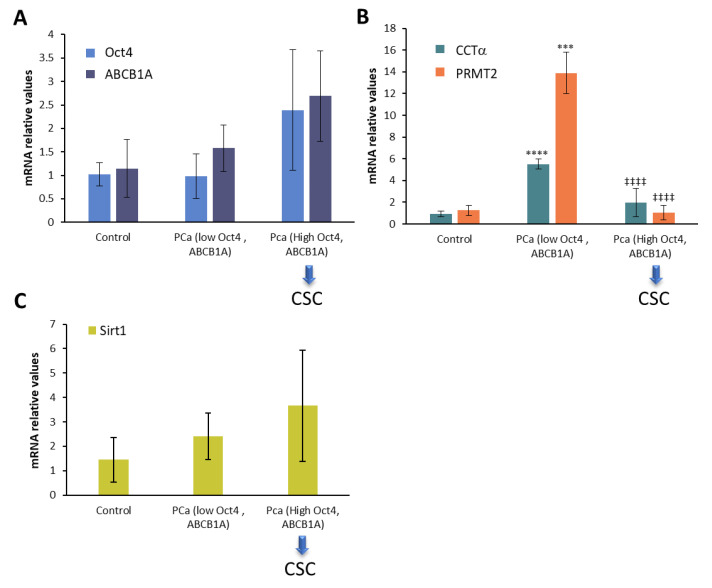
mRNA levels of CTP:choline cytidylyltransferase (CCTα), protein arginin N-methyl transferase 2 (PRMT2), and Sirtuin1 (Sirt1) in human prostate cancer biopsies determined via qPCR. (**A**) Levels of Oct4 and ABCB1A were determined via qPCR using 18S as a housekeeping gene. Based on the expressions of these genes, prostate biopsies were stratified into three groups: control (*n* = 5), prostate cancer (PCa) low Oct4 and ABCB1A (*n* = 7), and PCa high Oct4 and ABCB1A (*n* = 8) (blue arrows). (**B**) CCTα and PRMT2 and (**C**) Sirt1 mRNA levels were measured in prostate biopsies via qPCR using Actin as a housekeeping gene. *** *p* < 0.001 and **** *p* < 0.0001 indicate significant differences between control and PCa biopsies via two-way ANOVA and Sidak’s multiple comparisons test. ^‡‡‡‡^ *p* < 0.0001 indicates significant differences between PCa low Oct4 and ABCB1A and PCa high Oct4 and ABCB1A biopsies.

Given that the drug-resistant prostate cancer cell line LN-FLU exhibited stem cell-like characteristics and the upregulated expressions of pluripotent genes such as OCT4, as well as efflux transporters like ABCB1A, we examined the expressions of the stem cell markers Oct4 and ABCB1A in the prostate cancer patient samples. Consequently, we identified a subset of biopsies showing elevated expressions of Oct4 and ABCB1A, indicating an enrichment in the cancer stem cells (Figure 6A, highlighted by blue arrow). Notably, in the biopsies in which stem cell gene expressions were increased, we observed a concurrent decrease in both the CCTα and PRMT2 levels (Figure 6B) and an increase in Sirt1 (Figure 6C), which were in agreement with those observed in the prostate cells. This intriguing finding led us to speculate that drug resistance may be associated with the reprogramming of cells into cancer stem-like cells, characterized by the suppression of phosphocholine metabolism and methylation and the upregulation of deacetylation.

These observations shed light on a potential link between altered cellular metabolism, epigenetic modifications, and the acquisition of drug resistance through the induction of cancer stem cell-like properties.

## 3. Discussion

Despite the significant progress made in cancer therapy, and the positive results of androgen-deprivation therapy in prostate cancer (PCa), the development of drug resistance and subsequent recurrence remains a significant challenge. In PCa, the androgen receptor (AR) plays a crucial role in supporting the metabolic and biosynthetic demands of cancer cells [31]. Therefore, it could be possible that androgen receptor antagonists drive any kind of adaptative response that allows the tumoral cells to survive in stressful conditions [32]. The adaptive program activated in response to such conditions aims to support cell survival and protect cells against the detrimental effects of stressors. For instance, it was observed that the in vitro, long-term treatment of prostate cancer cells with enzalutamide up to 5 µmol/L induced drug resistance and prolonged AR signaling suppression, as well as the downregulation of the cell cycle, Wnt signaling, and DNA repair pathways [33]. Accordingly, our results show a downregulation of the AR and an inhibition of the Akt/mTOR (mammalian target of rapamycin) pathway. Akt is a serine/threonine kinase that plays a crucial role in promoting cell survival and growth. It is activated through phosphorylation, which leads to the activation of the downstream signaling pathways involved in cell proliferation and survival. The decrease in the phosphorylation of Akt and mTOR observed in our work suggests a dampened activation of these pathways, which could have inhibited the growth of the drug-resistant LN-FLU cells. Similarly, mTOR is another key protein involved in regulating cell growth and proliferation. The activation of mTOR signaling promotes protein synthesis, cell growth, and proliferation. In the drug-resistant LN-FLU cells, the decreased levels of mTOR indicate a potential disruption in this signaling pathway, which can further contribute to the reduced proliferation and growth observed in these cells. Furthermore, our results show that the resistant LN-FLU cells have reduced expressions of Cyclin D1 and increased levels of the cyclin inhibitor p16 compared to the parental LNCaP cells, suggesting a cell cycle arrest, indicative of a non-proliferating state compatible with cell dormancy [34]. This is in agreement with the group of Aguirre-Ghiso, who proposed that perhaps the dormancy of tumor cells is an active program that recapitulates the quiescence of normal stem cells [35]. Moreover, in a clinical trial with 36 prostate cancer patients, it was observed that low androgen receptor transcriptional activity and higher stemness were tightly linked to a failure in the enzalutamide response [36], which is in good agreement with our results. 

Dormancy has been considered an adaptation mechanism that allows cells to survive in hostile conditions [21]. Dormant cells can be non-cycling or slow cycling and can exhibit stem-like properties. This state of dormancy is believed to play a significant role in cancer progression and treatment resistance, as dormant tumor cells have the ability to survive and eventually resume growth, leading to disease recurrence or metastasis [37]. Previous data suggest that dormant disseminated tumor cells not only undergo a deep growth arrest but also exhibit an upregulation of genes associated with self-renewal and pluripotency, including Sox9, Oct4, Sox2, and Nanog [38]. These observations indicate that dormant cancer cells share characteristics with both embryonic and adult stem cells, and it implies a relationship between cell dormancy and cancer stem cells. Indeed, cancer cell dormancy can be considered as an epigenetic state in which cells remain quiescent while preserving their self-renewing capacities. Our data show that LN-FLU cells upregulate the expressions of the canonical stem cell markers CD133 and ALDH1A1 and the transporter ABCB1A, also named P glycoprotein, a well-known member of the multi-drug-resistant protein family that effluxes xenobiotics outside the cell, thereby contributing to drug resistance. Furthermore, our results also show an increase in the pluripotent transcription factors Oct4 and Nanog in the resistant LN-FLU cells, further confirming the stemness of the resistant quiescent LN-FLU cells. Our results are in good agreement with a previous study that observed a marked upregulation of genes associated with cancer stem cells, such as Oct4, Sox2, Nanog, BMP2, and ALDH1, in prostate enzalutamide-resistant cells [33].

In the dormant state, disseminated tumor cells exhibit a reduced metabolic activity and proliferation rate, allowing them to evade detection and remain undetected for prolonged durations [39]. In addition, in stressful conditions, such as exposure to chemotherapy or other forms of cellular stress, alterations in phosphatidylcholine (PC) metabolism can occur as part of an adaptive program. Moreover, it has been described that phosphocholine and phosphatidylcholine metabolism are closely related to leukemia patients’ survival [40]. Our results demonstrating a decrease in the enzymes involved in the phosphatidylcholine biosynthetic Kennedy pathway indicated a depletion of the choline metabolites involved in this pathway (i.e., phosphocholine, CDP-choline, and phosphatidylcholine) in the stem-like drug-resistant LN-FLU cells, further suggesting a dormant state that survives in low metabolic activity. This is in agreement with a previous study of 31 prostate cancer patients who underwent radical prostatectomy, in which the authors showed that the plasma lysophosphatidylcholine (LPC) concentration was significantly more decreased in cancer tissues than in normal tissues [26]. This suggests that, under specific circumstances, there could be a connection between prostate cancer and a depletion in phosphatidylcholine metabolism. Our observations of prostate cancer biopsies demonstrate that the reduction in PC metabolism is linked to an increased presence of cancer stem cells, potentially leading to drug resistance. 

Our results also show a modification of the enzymes involved in epigenetic regulation: PRMT2 and Sirt1. Sirt1 is a NAD+-dependent deacetylase, closely related to starvation conditions. In addition, a significant role in various facets of cancer drug resistance has recently been attributed to Sirt1 [41]. Recently, Sirt1 has been implicated in the extension of the life span, promoting longevity in various organisms, including yeast, worms, flies, and mice [42,43]. In addition, the phosphorylated active form of Sirt1 has been implicated in replication origin dormancy, preventing the recruitment of the DNA damage response kinase ATR to chromatin [44]. Here, we show that long-term treatment with the antiandrogen FLU activates Sirt1 and decreases the expression of PRMT2, suggesting that LN-FLU cells may undergo specific epigenetic changes that enable them to enter a dormant state and persist in a non-proliferative state that contribute to the ability of cancer cells to evade therapies. Our results reinforce the notion that the generation of dormant resistant cells may be promoted via chemotherapy [12], and therefore cancer therapy may induce the surviving residual tumor cells to enter a dormant state by activating stress signaling pathways, as previously considered [35]. This notion also agrees with studies by Li et al. showing that the short-term exposure of 10 days of tumor prostate cells to chemotherapy at clinically relevant doses enriched a dormant tumor cell population [45]. In a relevant study by Lee et al., the authors investigated organoids obtained from a patient with metastatic prostate cancer and cultured them with an anti-androgen therapy. The findings of their research revealed that the organoids developed resistance to the therapy and showed an upregulation of neurogenic genes [46]. Additionally, the study demonstrated that treatment with enzalutamide induced a gradual transition of the organoids into a reversible dormant state. These findings are in strong alignment with the results of our own research, further supporting the notion that the development of therapy resistance and the induction of a dormant state are interconnected phenomena in the context of prostate cancer treatment.

Therefore, it is plausible that the cellular stress response triggered by cancer therapy may, in some cases, drive a subset of tumor cells into a dormant state as a survival mechanism. These dormant cells, while temporarily resistant to treatment, can later emerge as a source of tumor recurrence or metastasis. Further research is needed to elucidate the specific mechanisms by which therapy-induced stress signaling may contribute to tumor cell dormancy and its subsequent implications for treatment outcomes, in order to develop innovative therapeutic approaches that can effectively prevent tumor recurrence and improve patient outcomes.

## 4. Materials and Methods

### 4.1. Patient Selection and Tissue Sampling

A total of 15 patients (median age of 68 years) undergoing surgical radical prostatectomy at Principe de Asturias University Hospital (HUPA) (Alcalá de Henares, Spain) were selected for this study. After the intervention, a pathologist determined the stage of prostate cancer, according to the Gleason classification [47]. Samples of prostate tissue, and of adjacent visually unaffected tissue, were acquired from the resected prostate within 15 min of surgery. Tumor tissues were cut by the pathologist and were taken from where the tumor was most vigorous, while the presumed normal tissues were taken from the resected prostate far away from the tumor. Tissue samples were frozen in liquid nitrogen and stored at −80 °C until analyzed. The study was approved by the HUPA Ethics Committee (PROCARE 2020) and the Alcalá University Ethics Committee (CEIP/HU/2021/2/036) and followed the principles outlined in the Declaration of Helsinki.

### 4.2. Cell Culture

The human prostate cancer cell lines LNCaP and PC3 (ATCC CRL-1740 and ATCC CRL-1435, respectively, ATTC, Rockville, MD, USA) were cultured in RPMI-1640/10% FBS supplemented with 100 IU/mL penicillin G sodium, 100 µg/mL streptomycin sulfate, and 0.25 µg/mL amphotericin B (Invitrogen, Paisley, UK). To establish the prostate cancer-resistant cell line, LNCaP cells were cultured for six months with a gradual increase in the concentration of the antiandrogen 2-hydroxy-flutamide. Once cells were able to grow in the presence of 2 µM 2-hydroxy-flutamide, they were maintained at that concentration and named LN-FLU.

### 4.3. Cell Viability

Cell viability was assayed via MTT assay. Cells (1.5 × 10^5^ cells/well) were seeded in 12-well plates. After 48 h, the cells were exposed to 2-hydroxi-flutamide (FLU) or docetaxel (DTX) for 24 h. Subsequently, 100 μL of MTT (Sigma-Aldrich, St. Louis, MO, USA) was added to each well and incubated at 37 °C for 1 h. Following the incubation, the medium was removed, and the formazan crystals were dissolved in 2-propanol. Optical density measurements were conducted at 595 nm using a microplate reader (iMARK, Bio-Rad Laboratories, Inc., Hercules, CA, USA). Cell viability was calculated as a percentage relative to the vehicle-treated sample, which was assigned a viability of 100%.

### 4.4. Cell Proliferation Assay with 5-Bromo-2′-deoxyuridine (BrdU)

LNCaP, LN-FLU, and PC3 cells were initially seeded in 12-well plates (1.5 × 10^5^ cells/well) with a glass coverslip per well. After 24 h, the culture medium was replaced with serum-free RPMI-1640 medium, and different treatments were added. During the last 16 h of treatment, 10 μM of 5-bromo-2′-deoxyuridine (BrdU) (Sigma-Aldrich, St. Louis, MO, USA) was added to each well. They were then subjected to 0.1 M sodium borate for 2 min at room temperature, washed twice with PBS, and subsequently exposed to 2 M HCl for 20 min to partially denature the DNA. Then, they were incubated with 0.1 M sodium borate for 2 min at room temperature, washed twice with PBS, and permeabilized with 0.2% Triton X-100 in PBS containing 3% bovine serum albumin for 5 min. The cells were washed twice with PBS and then incubated with a monoclonal anti-BrdU antibody (BD Biosciences, San Diego, CA USA) (1:50) at 37 °C for 2 h. They were washed twice with PBS and then incubated with mouse Alexa Fluor 488-conjugated secondary antibody (Invitrogen, Carlsbad, CA, USA) (1:2000) and DAPI (Sigma-Aldrich, St. Louis, MO, USA) (1:2000) for 1 h at 37 °C. Finally, cells were washed twice with PBS and then mounted with Mowiol mounting medium (Sigma-Aldrich, St. Louis, MO, USA). The images were acquired using a Leica DM100 microscope with Leica LAS X V4.6 Imaging software (Leica Microsystems, Wetzlar, Germany, https://www.leica-microsystems.com/es/productos/software-de-microscopia/p/leica-las-x-ls/, accessed on 22 October 2023) and an oil immersion objective of 63×.

### 4.5. Cell Proliferation Assay with Carboxyfluorescein Succinimidyl Ester (CFSE)

LNCaP, LN-FLU, and PC3 cells were labeled with 2.5 µM BioTracker™ 488 Green carboxyfluorescein succinimidyl ester (CFSE) Dye (CS224588) according to the manufacturer’s instructions for the BioTracker™ 488 Green CSFE Cell Proliferation Kit (SCT110, Sigma-Aldrich, St. Louis, MO, USA). Then, cells were seeded in 12-well plates (2 × 10^5^ cells/well). After 24 h of growth, cells were treated for 72 h in complete medium (RPMI-1640 10% FBS 1% Antibiotic) to avoid cell cycle arrest. Data acquisition and analysis were performed in a MACSQuant^®^ analyzer flow cytometry system (Miltenyi Biotec, Bergisch Gladbach, Germany) using the MACSQuantify™ software (Miltenyi Biotec, Bergisch Gladbach, Germany, https://www.miltenyibiotec.com/ES-en/products/macs-flow-cytometry/software/macsquantify/flow-cytometry-software.html, accessed on 22 October 2023). A total of 10 × 10^3^ events were collected for each sample.

### 4.6. Western Blot

2 × 10^6^ cells were lysed in 100 μL Lysis Buffer (50 mM Tris pH 7.4; 0.8 M NaCl; 5 mM MgCl_2_; 0.1% Triton X-100) containing a protease inhibitor and phosphatase inhibitor cocktail (Roche, Diagnostics; Mannheim, Germany) and incubated for 15 min at 4 °C. Insoluble fragments were removed via centrifugation at 10,000× *g* for 5 min at 4 °C. Protein concentration in the supernatant was determined using the Bradford Protein Assay Kit (BioRad, Hercules, CA, USA). Twenty micrograms of total protein was loaded, separated on 8% SDS-PAGE gels, and transferred onto PVDF membranes. Membranes were incubated overnight at 4 °C with the primary antibodies (Table 2). After washing the membranes three times in TBS, they were incubated with horseradish peroxidase-conjugated anti-mouse or anti-rabbit secondary antibodies (1:5000) for 2 h at room temperature. Finally, immunoreactions were visualized via an ECL system (Cell Signaling Technology, Danvers, MA, USA). Protein expression levels were quantified using ImageJ (National Institutes of Health, Bethesda, MD, USA, https://imagej.nih.gov/ij/download.html, accessed on 22 October 2023) and expressed as fold changes relative to the control treatment. All Western blots were repeated at least three times.

### 4.7. RNA Extraction and Reverse Transcription Quantitative Polymerase Chain Reaction

Cellular RNA was isolated from cells using the NZT Total RNA Isolation kit (Nzytech, Lisbon, Portugal). A total of 2 μg of RNA was subjected to cDNA synthesis using the NZY First-Strand cDNA Synthesis Kit (Nzytech, Lisboa, Portugal), following the manufacturer’s instructions. Quantitative PCR (qPCR) was performed in a 10 μL reaction volume using the NZY First-Strand cDNA Synthesis Kit (Nzytech, Lisboa, Portugal). The PCR amplification utilized the primer sequences shown in Table 3.

### 4.8. Fluorescence Microscopy

Cell samples were subjected to fixation in a phosphate-buffered saline (PBS) solution containing 4% paraformaldehyde, followed by treatment with 0.1% Triton X-100 to allow for permeabilization. Immunolabeling with an anti-CTTα antibody (Cell Signaling Technology, Danvers, MA, USA) was performed via incubation at room temperature for 1 h. Then, a secondary labeling approach utilized secondary antibody conjugated with Alexa Fluor 488 from Invitrogen (Carlsbad, CA, USA) and DAPI (Sigma-Aldrich, St. Louis, MO, USA). Finally, cells were mounted onto coverslips using a Mowiol mounting medium and examined under a Leica TCS SP5 laser-scanning confocal microscope, equipped with Leica LAS-AF imaging software (Leica Microsystems, Wetzlar, Germany), and a 63× oil objective.

### 4.9. Analysis of Phosphatidylcholine Levels 

An amount of 2 × 10^6^ LNCaP and LN-FLU cells was seeded on P100 plates and the phosphatidylcholine (PC) levels were determined using the Phosphatidylcholine Assay Kit (MAK049, Sigma, St. Louis, MO, USA) following the manufacturer’s instructions. In this assay, the PC concentration is determined via a coupled enzyme reaction, which results in a fluorometric (λex = 535/λem = 587 nm) product, proportional to the PC present. A standard curve was prepared, and the final amount of phosphatidylcholine was found by dividing the nmoles obtained by the amount of total protein for each sample.

### 4.10. Statistical Analyses

The statistical analysis of the results was performed with Graph Pad Prism 9 software (San Diego, CA, USA) using a two-way ANOVA and Tukey’s multiple comparisons test or Sidak’s multiple comparisons test. The results are reported as the mean ± standard deviation (SD), as indicated in the figure captions, of at least three independent experiments. Data were considered significant when *p*  ≤  0.05.

## 5. Conclusions

Understanding the metabolic changes linked to chemoresistance in prostate cancer is essential and could potentially enhance treatment strategies. Our study shows that the long-term treatment of androgen-sensitive prostate cancer cells with the antiandrogen 2-hydroxy-flutamide leads to the development of a quiescent state with stem cell-like characteristics, enabling survival in a low metabolic state and conferring drug resistance. In this context, there is a reduction in the de novo biosynthesis of phosphatidylcholine and the activation of epigenetic reprogramming. 

## Figures and Tables

**Table 1:** Baseline characteristics of the prostate cancer patients.

	%	Mean ± SD	Median
Age (years)			
50–59	12.5	55.5 ± 0.7	55.5
60–69	62.5	66.4 ± 2.9	68.0
>70	25.0	73.5 ± 2.3	74.5
Weight (kg)			
60–79	50.0	72.0 ± 5.3	74
80–99	31.25	87.8 ± 5.1	86
>100	18.75	107.6 ± 5.8	110
BMI (kg/m^2^)			
20–25	18.75	23.4 ± 0.5	23.7
26–30	50.0	27.6 ± 1.4	27.25
>30	31.25	33.8 ± 3.0	34.1
Gleason Grade			
6–7	26.6	6.5 ± 0.5	6.5
7–8	60.0	7.3 ± 0.5	7
>8	13.4	9 ± 0.0	9
PSA (ng/mL)			
1–7	50	4.9 ± 1.8	4.74
7–20	31.25	9.1 ± 2.2	8.12
>20	18.75	28.4 ± 12.6	21.22

A total of 19 prostate cancer patients scheduled for surgical radical prostatectomy were selected for this study. Age values (in years), weight (in kg), Body Mass Index (BMI) (in kg/m^2^), Gleason grade of the piece, and the prostate-specific antigen (PSA) (in ng/mL) are shown in the columns. Above, in bold, are percentage of individuals, mean value ± SD (standard deviation), and median of each group.

**Table 2:** Antibodies and dilutions used in the Western blots.

Antibody	Dilution	Reference and Company
AR	1:500	sc-7305; Santa Cruz Biotechnology (Dallas, TX, USA)
pAkt (Ser 473)	1:1000	#4060; Cell Signaling Technology, Danvers, MA, USA
Akt	1:1000	#4691; Cell Signaling Technology, Danvers, MA, USA
pmTOR	1:1000	#2971; Cell Signaling Technology, Danvers, MA, USA
mTOR	1:1000	#2972; Cell Signaling Technology, Danvers, MA, USA
Cyclin D1	1:1000	#RM-9104-S0; Invitrogen, Paisley, UK
p16	1:500	sc-467; Santa Cruz Biotechnology (Dallas, TX, USA)
CD133	1:500	#64326; Cell Signaling Technology, Danvers, MA, USA
ALDH1A1	1:500	#12035; Cell Signaling Technology, Danvers, MA, USA
CCTα	1:1000	#6931; Cell Signaling Technology, Danvers, MA, USA
CK	1:1000	#13422; Cell Signaling Technology, Danvers, MA, USA
PRMT2	1:500	ab154154; Abcam, Cambridge, UK
pSIRT	1:500	#2314; Cell Signaling Technology, Danvers, MA, USA
SIRT	1:500	#9475; Cell Signaling Technology, Danvers, MA, USA
β-actin	1:5000	A5441; Sigma, St. Louis, MO, USA
Rabbit (HRP)	1:5000	#7074S; Cell Signaling Technology, Danvers, MA, USA
Mouse (HRP)	1:5000	A-9044; Sigma, St. Louis, MO, USA

**Table 3:** Primer sequences for PCR amplification.

Gene	Forward	Reverse
*Oct4*	5′-GACAGGGGGAGGGGAGGAGCTAGG-3′	5′-CTTCCCTCCAACCAGTTGCCCCAAAC-3′
*Nanog*	5′-TTTGTGGGCCTGAAGAAAACT-3′	5′-AGGGCTGTCCTGAATAAGCAG-3′
*ABCB1A*	5′-TTGCTGCTTACATTCAGGTTTCA-3′	5′-AGCCTATCTCCTGTCGCATTA-3′
*PEMT*	5′-CTCTAAGCGTCACCATCCTGCT-3′	5′-GGTGTCCAGGCTCTCCATCCT-3′
*PRMT2*	5′-GCAGTTGGACATGAGAACCGTG-3′	5′-AGGCTCTGGAAGTGGACGCTAA-3′
*Sirt1*	5′-ACCCAGAACATAGACACGCTGGAA-3′	5′-TCTCCTCGTACAGCTTCACAGTCA-3′
*CCTα*	5′-GTGGTCATTACAGACCCTGAAAA-3′	5′-AACTCTTCTAACTGCCATAGCAC-3′
*Actin*	5′-AGAAGGATTCCTATGTGGGCG-3′	5′-CATGTCGTCCCAGTTGGTGAC-3′

## Data Availability

The datasets and/or analyses used during the current study are available at https://doi.org/10.17632/mkv5vjkvjt.1, accessed on 25 August 2023.

## Supplementary Materials

The supporting information can be downloaded at: https://www.mdpi.com/article/10.3390/ijms242115626/s1.
