# Supplementary figures and images for: Resistance to 2-Hydroxy-Flutamide in Prostate Cancer Cells Is Associated with the Downregulation of Phosphatidylcholine Biosynthesis and Epigenetic Modifications
Source: Int J Mol Sci. 2023 Oct 26;24(21):15626. doi: 10.3390/ijms242115626 (PMC10650717; doi:10.3390/ijms242115626)

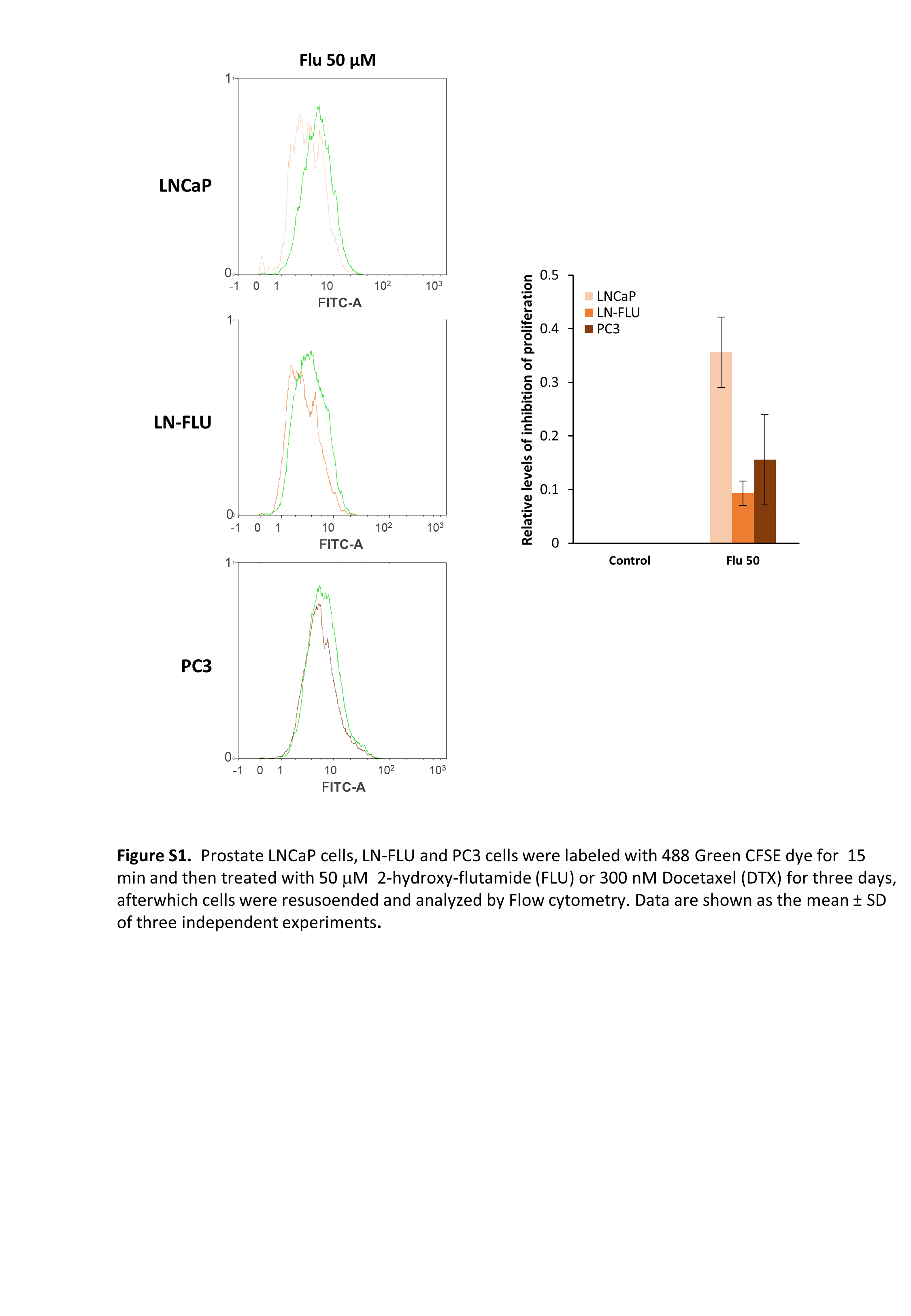

Supplement: Supplementary file 1 [file ijms-24-15626-s001.zip › ijms-2594598-supplementary.tif]
